# Acute flaccid myelitis in low- to middle-income countries: diagnosis and surveillance

**DOI:** 10.1093/braincomms/fcae167

**Published:** 2024-05-15

**Authors:** Jelte Helfferich, Amary Fall, Carlos A Pardo, Bart C Jacobs, Kevin Messacar

**Affiliations:** Department of Neurology, University Medical Center Groningen, University of Groningen, 9700 RB Groningen, The Netherlands; Division of Medical Microbiology, Department of Pathology, Johns Hopkins University School of Medicine, Baltimore, MD 21205, USA; Department of Neurology, Johns Hopkins University School of Medicine, Baltimore, MD 21287, USA; Department of Neurology and Immunology, Erasmus MC, University Medical Center Rotterdam, 3015 CN Rotterdam, The Netherlands; Department of Pediatrics, Children's Hospital Colorado, University of Colorado, Aurora, CO 80045, USA

With interest, we have read the article by Olum *et al.*^1^ published recently in *Brain Communications*. In their report, they describe six cases of suspected acute flaccid myelitis (AFM), identified in a relatively short time in Uganda. They discuss the difficulty in confirming the diagnosis without the availability of additional investigations and the limitations this produces in the applicability of the current diagnostic criteria in low- to middle-income countries (LMIC). Together with the limitations in infrastructure for adequate surveillance, this has implications for the estimation of the global incidence and health impact of AFM.

## Clinical diagnosis

In children presenting with acute flaccid paralysis (AFP), it may be difficult to make an aetiological diagnosis at onset, even if all investigations, including imaging and virological tests, are available.^2,3^ Contrary to poliomyelitis, where the diagnosis can be confirmed by finding poliovirus in a faecal sample of a patient with AFP, the diagnosis of AFM depends on clinical and diagnostic characteristics, as different viruses other than poliovirus may be associated.^4^ Early clinical features that may help to differentiate AFM from other causes of AFP in children have been identified (Table 1).^2,3,5^ These include the presence of a sensory level, described in one of the cases by Olum *et al*., which would, in our opinion, exclude the diagnosis of AFM.^1^ AFM is mainly an anterior horn disease, while a sensory level indicates more diffuse spinal cord involvement, which may be present in other forms of paediatric myelopathies such as neuromyelitis optica and myelin oligodendroglial glycoprotein (MOG) antibody-associated disease or acute vascular injury of the spinal cord. The features presented in Table 1 may also be helpful in determining the cause of AFP in areas where investigations are limitedly available. Recurrence of weakness is included in this table as being atypical for a diagnosis of AFM, especially with the described interval of more than 6 months, as discussed by the authors.

**Table 1:** Early features that may help to make an early diagnosis of AFM in clinical practice

Supportive features for the diagnosis of AFM	Features that argue against the diagnosis of AFM
**Decreased or absent reflexes in affected limbs**	**Hyperreflexia in affected limbs**
**Predominantly proximal weakness**	**Predominantly distal weakness**
**Asymmetric weakness**	**Symmetric weakness**
**Prodromal (respiratory) illness**	**Predominant bladder and bowel dysfunction**
**Time course from prodrome till onset of < 7 days**	**Time course from onset till nadir >10 days**
**Pleocytosis in CSF (>5 leukocytes/μL)** ^a^	**Sensory deficits/sensory level**
	**Significantly raised CSF protein level, especially in the absence of pleocytosis recurrence**
MRI lesions with predominant grey matter involvement	Absence of MRI abnormalities in spinal cord
	Isolated conus involvement on spinal cord MRI
	Supratentorial abnormalities on brain MRI
Features suggestive for axonal damage on NCS/EMG	demyelinating features on NCS
EV-D68 or other associated enterovirus in any material	
	MOG/AQP-4 antibodies in serum

The features included in Table 1 are based on findings from different studies. The upper part of the table, in bold, includes items applicable countries with limited resources. The items in the lower part may not be feasible for these countries but do provide additional support for the diagnosis.

^a^A mild increase of leucocyte cell count (5–50/μl) is seen in 15% of patients with GBS.

EMG, electromyography; NCS, nerve conduction studies.

Importantly, while MRI may not be available in many countries, the clinical profile along with basic cerebrospinal fluid (CSF) investigations during the acute phase, which are generally easily accessible in the majority of LIMC, would improve the certainty of the diagnosis of AFM. A comprehensive study of AFM cases during the 2018 outbreak showed that 92% of cases were preceded by prodromal fever or respiratory infection symptoms and 87% exhibited increased pleocytosis in CSF during the acute phase.^6^ CSF studies may provide important clues in differentiating AFM from non-inflammatory causes of acute spinal cord injury, such as spinal cord ischaemia.^7^ Combined with clinical signs such as asymmetry and slower progression of weakness, CSF outcomes may also help to differentiate AFM from Guillain–Barré syndrome, a frequent cause of AFP (Table 1).^5^ Therefore, of the available tests in LMIC, CSF investigations could be of particular value. Findings from basic nerve conduction studies (NCS) may also support the diagnosis, but these do require specific equipment and neurology expertise, which may not always be present.

Lastly, after the exclusion of poliovirus, the identification of another virus associated with AFM from the testing of biological specimens provides important support for the diagnosis of AFM. The time dependency of obtaining early specimens for virological testing and restricted ability to conduct further typing do pose limitations, but the central laboratories involved in AFP surveillance established by the WHO, which involve testing for poliovirus, might be able to help, as is indicated by a study performed in several countries in West Africa.^8^

### Diagnostic criteria

As the authors properly indicate, MRI for imaging confirmation of myelitis of the grey matter of the spinal cord is an essential part of the current diagnostic criteria of AFM.^4^ This has two important consequences for patients with suspected AFM: (i) if an MRI is not obtained, the diagnosis will remain uncertain and (ii) if an MRI does not show abnormalities, the diagnosis will be excluded.

The first consequence is important for countries with limited availability of MRI such as Uganda, as a possible, probable, or definite diagnosis AFM cannot be made. The second is also important for high-income countries, as abnormalities may be very difficult to detect, which might lead to incorrect rejection of the diagnosis.^3^ We do, however, believe that, when available, detection of grey matter abnormalities on MRI is important for confirming the diagnosis of AFM and maybe even more so for excluding alternative causes of AFP in clinical practice, similar to the situation for other inflammatory myelopathies.^7^

The strictness of diagnostic criteria largely depends on their purpose, which could be clinical diagnosis, application for research or for surveillance. For research purposes, for example to describe the clinical features of a disease, pathogenic mechanisms, or to investigate the effect of certain treatments, the diagnosis should be as certain as possible, which requires strict criteria. In the case of AFM, MRI abnormalities in the grey matter amongst other features need to be included.^9^

In clinical practice, a certain level of uncertainty is not uncommon, and the management of a patient needs to be done based on the most probable diagnosis. Therefore, if the diagnosis of AFM seems most probable relative to other causes (supported by the features in Table 1), the appropriate management and observation can be started, even if the diagnosis is uncertain according to the diagnostic criteria.

Olum *et al*.^1^ propose a revision of the clinical criteria, in which they suggest including (i) the absence of sensory involvement as a supportive feature and (ii) development of spasticity or hyperreflexia at follow-up as a feature excluding AFM. We agree that these features indicate other diagnoses. In fact, the presence of sensory abnormalities is already included as a feature suggesting an alternative diagnosis in the current criteria.^4^ Spasticity or hyperreflexia at follow-up is useful for a delayed diagnosis or surveillance if follow-up can be achieved but cannot be used in the acute phase when decisions about management need to be made. We propose that Table 1, which includes the features suggested by the authors, can be used for a clinical diagnosis in resource-poor settings.

### Surveillance

We underline the importance of gaining insight into the global incidence of AFM and its burden of disease. Currently, incidence numbers are largely unknown. Data from LMIC are very limited, but there is also a paucity of data from many high-income countries.^10^ Surveillance for AFM is only performed in some countries, and different strategies are used. These include surveillance based on clinical criteria and surveillance based on associated pathogens. The latter is important to determine the association with different pathogens and gain insight into pathophysiology and aetiology, but this approach requires adequate and early virologic tests and facilities for viral genomic studies.

While resources for genomic studies have greatly improved in LMIC during the COVID pandemic, clinical surveillance seems to be more feasible. However, the current case definitions, requiring MRI, are not practicable. Therefore, there is a high need for a case definition which can also be applied in LMIC. As AFP is still a reportable condition in many of these countries, mainly to detect cases of poliomyelitis, the infrastructure of the AFP surveillance may provide a basis for AFM surveillance. To move forward in recognizing, detecting, and managing this debilitating condition, it is essential to raise awareness amongst clinicians in LMIC and collaborate globally with inclusion of healthcare providers from LMIC in the international AFM working group.

## Competing interests

None declared. Views expressed are solely those of the authors and do not represent official positions of their institutions or committees with which they are affiliated.

## Data Availability

Data sharing is not applicable to this article as no new data were created or analysed.
